# RHOX Homeobox Transcription Factor Regulation of *Ins2* in Rodent Granulosa Cells

**DOI:** 10.3390/cells14070478

**Published:** 2025-03-22

**Authors:** Kanako Hayashi, James A. MacLean

**Affiliations:** 1 Center for Reproductive Biology, School of Molecular Biosciences, Washington State University, Pullman, WA 99164, USA; k.hayashi@wsu.edu; 2 Department of Physiology, Southern Illinois University, Carbondale, IL 62901, USA

**Keywords:** ovary, granulosa cells, insulin regulation, homeobox genes

## Abstract

The *Rhox* family of homeobox transcription factors comprises established regulators of gonad function, but their downstream targets have been relatively elusive, particularly in the female reproductive tract. Here, we characterize *Ins2* as a downstream target of the two granulosa cell-specific factors, *Rhox5* and *Rhox8*, in the ovary. While INS2 is classically produced by islet cells in the pancreas, we found that *Ins2* gene expression is present in the mural granulosa cell layer of large antral follicles, and it was not significantly reduced in *Rhox5*-null mice. This was a surprising finding as we previously validated *Ins2* as a direct target of RHOX5 in Sertoli cells, the male counterpart to granulosa cells that serves the germ cell nurse function in the testis. In the ovary, RHOX8 appears to be the major driver of *Ins2* expression, as evidenced from the maximal activity of *Ins2* promoter reporter plasmids when RHOX8 protein was active within granulosa cells in vitro and the downregulation of endogenous *Ins2* in mice with the granulosa cell-specific knockdown of RHOX8 in vivo. RHOX5 induces *Rhox8* expression in pre-antral granulosa cells and then becomes relatively silent in peri-ovulatory follicles. However, *Rhox8* does not peak until after the ovulatory LH surge. The induction of *Rhox8* by progesterone, after the normal window of RHOX5 has passed, may explain why *Rhox5*-null female mice display apparently normal fertility, if RHOX8 is capable of the redundant stimulation of target genes that are essential for ovulation.

## 1. Introduction

Successful ovulation requires the coordinated expression of genes that must be turned on and off in the right place at the right time for proper follicle development [1,2]. During follicle growth, the developing oocyte is surrounded by granulosa cells that serve as nurse cells to support its maturation and growth, and the oocyte with an absence of granulosa cell signals leads to ovulation failure [3,4]. This is achieved in part by secreted hormones and growth factors from mural granulosa cells located in the outer wall of the follicle that act in a paracrine fashion on cumulus granulosa cells, which are immediately adjacent to the oocyte. In addition, the direct transfer of signals from the cumulus granulosa cells to the developing oocyte is possible. While the hormone signals from the pituitary that initiate ovulation are well characterized, the master control genes that regulate follicle growth within the ovary are not as well known. Homeobox transcription factors (e.g., *Hox* genes) are master regulators of developmental programs and at least 35 homeobox genes are known to be expressed in the ovary. Studies have clearly demonstrated an essential role for *Nobox* and *Lhx8* in ovulation [5,6,7], and the *Iroquois* and *Obox* homeobox gene clusters are differentially regulated during oocyte development, implying that they may be similarly important [8,9,10,11]. However, all of these factors are oocyte-specific; herein, we describe two granulosa cell-produced homeobox transcription factors that are candidates to regulate follicular growth and ovulation.

Defects in cellular metabolism such as disrupted insulin signaling in diabetic individuals are known to negatively impact fertility in rodents and primates [12]. Insulin II (*Ins2*) is one of two insulin genes in the mouse genome. While the *Ins1* and *Ins2* genes are best known for being expressed by islet cells in the pancreas, where they are expressed in a 1:2 ratio in the adult mouse pancreas [13]. However, one or both are also known to be expressed in the adult thymus and brain, as well as some embryonic organs and the yolk sac [14,15]. We and others have recently demonstrated the expression of *Ins2*, but not *Ins1*, mRNA in the testes [16,17]. The mouse INS2 protein possesses the ability to strongly promote glucose uptake and protein synthesis and is considered the primary metabolic hormone in mice [18,19]. In support of this, the Akita mouse is a diabetic mouse model that possesses a point mutation in *Ins2* that results in the accumulation of misfolded INS2 protein that cannot activate insulin receptors and ultimately causes the destruction of insulin-producing islet cells in the pancreas [20]. Interestingly, the Akita mouse exhibits a male subfertility phenotype that can be rescued by exogenous insulin treatment [17]. Female Akita mice and mice with streptozotocin-induced destruction of INS-producing pancreatic cells exhibit defects in oocyte maturation, development, and granulosa cell growth and survival, which lead to suboptimal ovulation and subfertility [21,22].

We became interested in the transcriptional control of insulin signaling when we discovered that RHOX5 directly regulates *Ins2* in mouse Sertoli cells [16]. *Rhox5* is the founding member of the reproductive homeobox X-linked (*Rhox*) gene cluster that encodes 13 distinct transcription factors in mice, with three genes having undergone tandem duplications to generate a cluster of 42 total genes [23,24]. Primates, rats, hamsters, dogs, cats, horses, sheep, and cattle possess *Rhox* gene orthologs (e.g., *RHOXF1*, *RHOXF2*) in the syntenic position on the X chromosome, although the composition of the cluster varies between species [25]. The function of most RHOX factors is unknown, but they are selectively expressed in the placenta, gonads, and reproductive tract. The majority of the cluster structure and expression data in non-rodent and primate species comes from genome project-related databases, and virtually no direct functional assays have been performed in vitro or in vivo. The majority of primate RHOX studies have focused on abnormal expression in cancerous tissues and putative roles in tumor establishment and progression in vitro using cell culture models and in genome-association studies with RHOXF1 and RHOX2 mutations [26,27,28]. Human RHOX factors are selectively expressed in oocytes and multiple germ cell types in the spermatogenic epithelium [29]. However, the only functional data for the human orthologs demonstrate roles in spermatogenesis, underlying complications in male fertility [24,30,31], and the protective suppression of LINE1 transposable elements in the male germline [32]. The relevance of RHOX-regulated pathways in the human ovary is, at present, unknown.

The most complete set of tools (molecular and genomic) to examine RHOX factor expression and function exists for rodents. Thus, our prior studies, and this one, have focused on analyzing the RHOX-dependent regulation of reproduction in mice. In the gonads, our analyses have demonstrated that only *Rhox5* and *Rhox8* are expressed in postnatal Sertoli and granulosa nurse cells [33,34,35,36]. The ablation of these genes results in male subfertility characterized by reduced spermatogenic output and motility defects [37,38]. Interestingly, *Rhox5*-null mice have a remarkably similar phenotype to that of Akita mice, suggesting that the RHOX5-regulation of *Ins2* may underly the Sertoli-cell defect in spermatogenesis. However, *Rhox5*-null female mice do not exhibit significant fertility complications, suggesting that if granulosa INS2 is essential for ovulation, other factors must contribute to its regulation [37]. *Rhox5* is induced by follicle-stimulating hormone (FSH) in granulosa cells and maintained by factors including GABP, SP1, CREB, and RAS signaling [33,35]. *Rhox5* contributes to maximal *Rhox8* induction in preantral follicles but is not required once luteinizing hormone (LH) and progesterone signaling are active [33]. We believe that ovulation is not severely compromised in *Rhox5*-null mice in part because RHOX8 expression is maintained in periovulatory follicles. In this report, we examine the regulation of the *Ins2* gene in ovarian granulosa cells by RHOX5 and RHOX8. Our findings demonstrate that *Ins2* expression is maintained at least in part by the RHOX8 stimulation of its promoter region. However, whether this regulatory relationship is the essential factor that spares *Rhox5*-null female mice from local metabolic or growth factor signaling defects that compromise ovulation remains to be determined.

## 2. Materials and Methods

### 2.1. Mice

All animal experiments were performed in accordance with the National Institutes of Health guidelines and in compliance with the Southern Illinois University Carbondale (protocols 16-043 and 19-007) and the Washington State University (protocols 6757 and 6767) Institutional Animal Care and Use Committees. The generation, genotyping, and characterization of *Rhox5*-null mice has been previously reported [16,37,39]. Conditionally activated *Rhox8* knockdown TARGATT mice were generated by Applied StemCell (Milpitas, CA, USA). *Amhr2*-Cre mice were provided by Richard Behringer (MD Anderson Cancer Center). All mice used in this study were maintained on a C57BL6 genetic background. All animals are housed under a 12:12 light–dark cycle at 70% humidity.

### 2.2. Plasmids and siRNA

To overexpress RHOX8, a full-length *Rhox8* coding sequence was amplified from total testis RNA. This 1022 bp product included in-frame restriction endonuclease recognition sequences to facilitate cloning into the pCDNA5/FRT vector (Invitrogen-Thermo Fisher, Waltham, MA, USA), which expresses recombinant genes under the control of the cytomegalovirus (CMV) promoter. For stable *Rhox8* expression, the pFRT/LacZeo plasmid was introduced in the genome of the rat spontaneously immortalized granulosa cell (SIGC) line. Subsequently, the *Rhox8* transgene was flipped into the FRT site from the pCDNA5/FRT:*Rhxo8* expression vector. Expression of *Rhox8* was confirmed by RT-PCR and immunofluorescence labeling of RHOX8 protein using a 1:2000 dilution of rabbit polyclonal NBP2-23671 (Novus Biologicals, Minneapolis, MN, USA), which we previously validated in the gonads [33,36]. The *Ins2* promoter parental reporter plasmid [40], deletion series, and RHOX binding site mutants were previously generated and characterized in our previous publication [16].

### 2.3. Superovulation and Granulosa Cell Cultures

For superovulation studies, female mice at postnatal age 21–28 days (PND21-28), selected by mass of at least 15 g for maximal response, were first injected with 5 IU equine chorionic gonadotropin (eCG; Biovendor RP178272, Ashville, NC, USA) as described previously [33,41]. Subsequently, mice were collected for the eCG-only group, or 48 h later, were given a single injection of 4 IU human chorionic gonadotropin (hCG; Sigma C0434, St. Louis, MO, USA). Mice were euthanized 2–24 h later and their ovaries were removed for histological or in vitro analyses. One ovary was homogenized in Trizol (Invitrogen-Thermo Fisher, Waltham, MA, USA) for RNA isolation and one ovary was fixed in 4% paraformaldehyde dissolved in PBS, pH 7.4 for 12–16 h then processed for embedding in paraffin.

Primary granulosa cells were isolated from eCG-primed mice as described previously [33,35]. Briefly, ovaries were removed and transferred to a 60-mm cell culture dish containing 5 mL of Dulbecco modified Eagle medium/F12 medium (DMEM/F12) supplemented with BSA, Fungizone, and gentamicin. Granulosa cells from multiple ovaries were pooled and treated with 20 µg/mL trypsin for 1 min, and then, 300 µg/mL soybean trypsin inhibitor and 160 µg/mL DNase I were added to remove necrotic cells. Cells were cultured at 37 °C in 95% air and 5% CO_2_ for 16 h before transfection. The SIGC line [42] was grown in DMEM-F12 medium supplemented with 5% FBS that was charcoal-stripped of hormones.

Transient transfection of both primary granulosa and SIGC was performed using the Attractene^TM^ transfection reagent (Qiagen, Germantown, MD, USA), which outperformed Lipofectamine 2000 (Invitrogen-Thermo Fisher, Waltham, MA, USA) and Turbofect (Fermentas-Thermo Fisher, Waltham, MA, USA), as assessed by cotransfection with green fluorescent protein (GFP) expression plasmids. Cell lysates were prepared and used to measure luciferase activity according to the manufacturer’s dual luciferase assay system protocol (Promega, Madison, WI, USA). Relative light units were normalized to the internal control plasmid pRL-TK and expressed as fold-change greater than that of the empty pGL3-basic vector. Transient knockdown of *Rhox8* was achieved by transfection of Qiagen’s FlexiTube GeneSolution GS434768 kit for *Rhox8*, which included a cocktail of four siRNA designed to block translation of RHOX8 and degrade *Rhox8* mRNA.

### 2.4. Real-Time Quantitative RT-PCR (qPCR) Analysis

Total RNA was isolated from ovaries using TRIzol reagent (Invitrogen-Thermo Fisher, Waltham, MA, USA), and then, RT was performed using High-Capacity cDNA Reverse Transcription Kit (Applied Biosystems, Waltham, MA, USA). The quantity and quality of RNA samples were determined by spectrometry and denaturing agarose gel electrophoresis, respectively. Real-time RT-PCR analysis of relative mRNA expression was performed on a MyiQ single-color real-time PCR detection system (Bio-Rad, Hercules, CA, USA) with iQ SYBR Green Supermix (Bio-Rad) according to the manufacturer’s recommendations. Real-time PCR was performed using the following protocol: 2 min at 95 °C, 40–45 cycles of denaturation (15 s at 95 °C) and annealing/extension (1 min at 60 °C), and a final step of melting curve analysis. As an internal control, *Rpl19* was used. The relative levels of mRNA were calculated using the 2^−ΔΔCt^ method. The primers for gene amplification have been previously reported and are included in Table 1 [33,36]. 

### 2.5. Immunohistochemistry

Immunolocalization of RHOX8 was performed in 5 µm cross-sections of paraffin-embedded ovarian tissue using a previously validated rabbit polyclonal antibody 2223B (Imgenex/Novus Biologicals, Centennial, CO, USA) at a 1:2000 dilution [33,38]. After washing, bound antibodies were visualized using Vectastain Elite ABC kit (Vector Labs, Newark, CA, USA) according to the manufacturer’s protocol. Negative control analyses were performed with preimmune serum provided by Lisa Stein (Imgenex, Newark, CA, USA), collected before the rabbit was immunized with the RHOX8 amino domain peptide.

### 2.6. In Situ Hybridization

Ovaries were sectioned at 5 µm and every 5th section was transferred to a microscope slide and stained with hematoxylin and eosin to assess morphology as previously described [13]. In situ hybridization analysis of *Ins2* mRNA expression was conducted using methods described previously [33]. Briefly, deparaffinized, rehydrated, and deproteinated sections were hybridized with radiolabeled sense or antisense cRNA probes generated from a linearized plasmid DNA template containing the entire *Ins2* coding sequence via incorporation of α-^35^S-uridine 5′-triphosphate. After hybridization, washing, and ribonuclease A digestion, slides were dipped in NTB liquid photograph emulsion (Kodak, Rochester, NY, USA), stored at 4 °C for 4–30 days, and developed in D-19 developer. Slides were then counterstained with Gill modified hematoxylin, dehydrated through ethanol series and xylene, and coverslipped. Regions of hybridization were visualized and localized by brightfield and darkfield microscopy.

### 2.7. Statistical Analysis

The qPCR expression time courses were subjected to one-way ANOVA Prism 9.0 (GraphPad, San Diego, CA, USA). Comparisons of means between two groups were conducted using student *t* tests and differences between individual means of multiple grouped data were tested by a Tukey multiple-range post-test. All data met the necessary criteria for ANOVA analysis, including equal variance as determined by Bartlett’s test. All experimental data are presented as mean ± SEM. Unless otherwise indicated, a *p* value of less than 0.05 was considered statistically significant.

## 3. Results

### 3.1. Ins2 Expression Tracks with Rhox8 Expression in the Ovary

**Figure 1:**
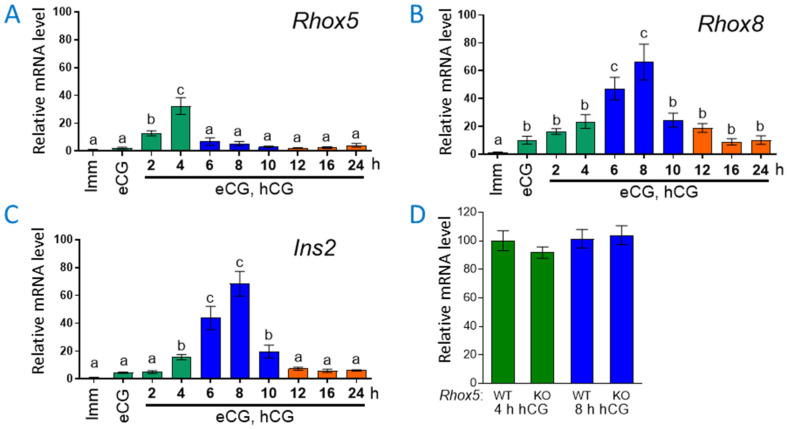
*Rhox8* and *Ins2* expression peak during the periovulatory window. (**A**–**C**) Immature C57BL/6 mice were superovulated with a single injection of 5 IU eCG followed by an injection of 4 IU hCG 48 h later. Intact ovaries were extirpated at 0, 2, 4, 8, 12, 16, and 24 h after hCG administration and RNA extracted. Relative expression levels were determined for each gene by qPCR [33]. Values (*n* = 6 animals per time point) were normalized against ribosomal L19 (*Rpl19*) mRNA and bars are shown as fold above background (±SEM), which was arbitrarily given a value of 1. Letters denote mean values that were significantly different (*p* < 0.05, one-way ANOVA with Tukey multiple-range post-test). Data are segregated into immature (purple), preovulatory (green), periovulatory (blue), and luteal (orange) phases of follicular development. (**D**) The expression of *Ins2* was assessed in ovaries from wild-type and *Rhox5*-null mice that were superovulated as described above and collected at 4 h and 8 h post-hCG. No significant differences in *Ins2* expression were observed (student’s *t* test) between *Rhox5*-null animals and wild-type (WT) animals. Data are presented as mean ± SEM relative mRNA expression where the WT values were set to 100% (*n* = 6 ovaries per time point and genotype).

As a first step towards examining whether RHOX5 and RHOX8 are candidates to regulate *Ins2* in the ovary as was previously found in the testis, we analyzed the time course of expression for each gene in total ovarian RNA obtained from hyperstimulated mice induced to ovulate with exogenous gonadotropins. The first injection of equine chorionic gonadotropin (eCG, which mimics FSH in mice) initiates an expanded wave of follicle growth and is followed by a second injection of human chorionic gonadotropin (hCG, which mimics LH) that elicits ovulation 10–12 h after hCG administration. In agreement with our prior studies [33,35], eCG induced the expression of *Rhox5* and *Rhox8*, with *Rhox5* peaking at 4 h post-hCG (Figure 1A) and *Rhox8* at 8 h post-hCG (Figure 1B). The expression profile of *Ins2* during ovulation has not previously been reported and we found that *Ins2* transcription was slightly induced by eCG but exhibited robust expression during the periovulatory window, similarly to *Rhox8* (Figure 1C). At 4 h post-hCG, *Ins2* mRNA was reduced in ovaries from *Rhox5*-null mice, but the decline was not significant (Figure 1D).

**Figure 2:**
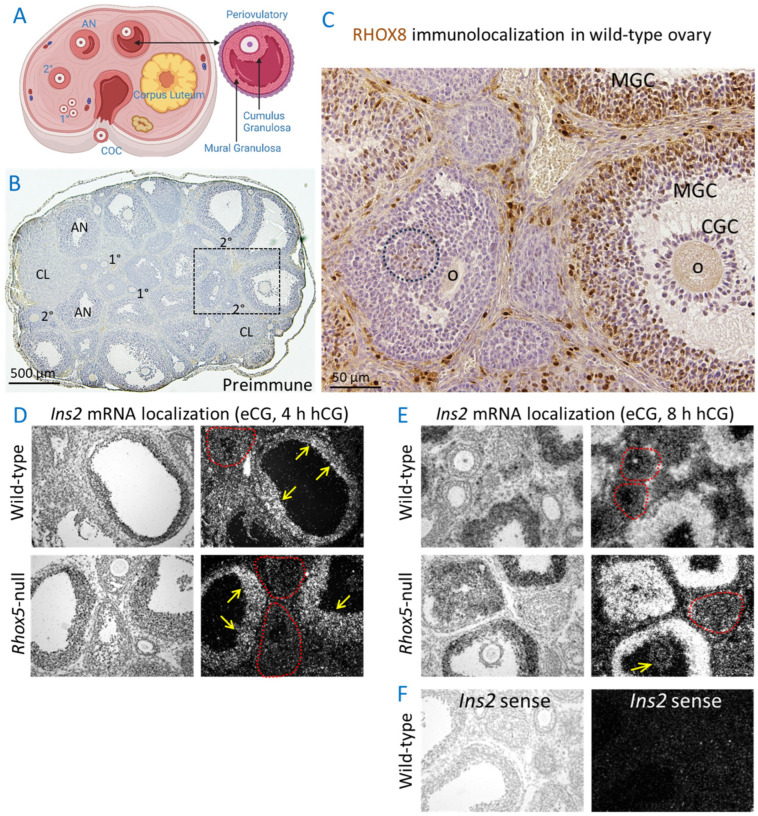
*Ins2* transcripts are localized to mural granulosa cells of antral follicles where RHOX8 is expressed. (**A**) Activated primary follicles (1°) are characterized by an oocyte with 1–2 layers of granulosa cells (GC); these rapidly grow to secondary follicles (2°) that contain multiple additional layers of GC. Once a fluid-filled cavity (i.e., antrum, AN) forms, the follicle is termed antral and continues to grow, segregating cumulus GC (CGC) stalk that supports the oocyte (O) and projects from the follicular wall where mural GC (MGC) are located. After ovulation and extrusion of the cumulus-oocyte complex (COC) the remaining GC differentiate into luteal cells within the corpus luteum (CL) that produce pregnancy-supporting hormones. Created in BioRender https://biorender.com/. MacLean, J. (https://biorender.com/t61b241, 12 March 2025). (**B**) All stages of follicular development are observed in wild-type ovaries, but no granulosa cells were detected with pre-immune serum from the rabbit used to make the RHOX8 antibody. (**C**) In contrast, RHOX8 protein is abundantly localized primarily in the MGC layer of antral follicles and is absent in the CGC layer. The region shown in this panel approximately corresponds to the outlined region in panel A, which was a serial section from the same block. The circle within the follicle transitioning from 2° to AN shows a few RHOX8-positive cells at this earlier developmental stage. Representative photomicrographs show that *Ins2* mRNA is present in antral follicles of superovulated mice at (**D**) 4 h post-hCG. Transcripts were abundant in the mural granulosa cell layer (yellow arrows) and absent in secondary follicles marked by red outlines. No differences between wild-type and *Rhox5*-null mice were observed. (**E**) *Ins2* expression increased at 8 h post-hCG but is not different between wild-type and *Rhox5*-null mice. The cumulus granulosa cell layer around the oocyte appeared to lack Ins2 transcripts (yellow arrow). (**F**) No appreciable signal was observed with the *Ins2* sense strand probe in any tissue section.

Primordial follicles in the ovarian stroma are recruited to grow and ovulate with each estrous cycle and transition from primary follicles (an oocyte surrounded by 1–2 layers of granulosa cells) to large antral periovulatory follicles that rupture to expel the cumulus oocyte complex (Figure 2A). As demonstrated in our prior report, only a few granulosa cells of primary and secondary follicles express RHOX8 [33], but RHOX8 protein is abundant in the mural granulosa cell layer of antral and periovulatory follicles (Figure 2C). While *Rhox5* transcription has waned before the periovulatory phase, RHOX5 protein is known to persist in about ~10% of mural granulosa cells [39], much lower than the proportion that express RHOX8. Commercially available RHOX5 antibodies do not react appropriately with the protein in tissues and the antibody that has been used previously is no longer available. Thus, no confirmatory immunolocalization could be performed.

We used radioactive in situ hybridization to determine the site and timing of *Ins2* transcription in ovarian follicles. The expression of the *Ins2* gene was robust enough to detect *Ins2* mRNA in ovarian follicles, primarily in the mural granulosa cell layer (Figure 2D, arrows). The expression of *Ins2* does not appear strong enough to be detected in preantral follicles (Figure 2D,E, red outlines). In agreement with our qPCR analysis (Figure 1D), there was no obvious reduction in *Ins2* mRNA in the granulosa cell layer of follicles in ovaries from *Rhox5*-null mice. At 8 h post-hCG, the upregulation of *Ins2* observed by qPCR was recapitulated in the mural granulosa cell layer (Figure 2E). However, *Ins2* expression in the cumulus granulosa cells layers was below the detection limit (Figure 2E, arrow). The expression levels of *Ins2* in periovulatory follicles of *Rhox5*-null mice were not obviously decreased relative to those of wild-type control mice. The localization of *Ins2* mRNA by the anti-sense probe was highly specific and no signals were detected with the negative control sense probe (Figure 2F).

### 3.2. Regulation of Ins2 in Spontaneously Immortalized Rat Granulosa Cells (SIGC)

**Figure 3:**
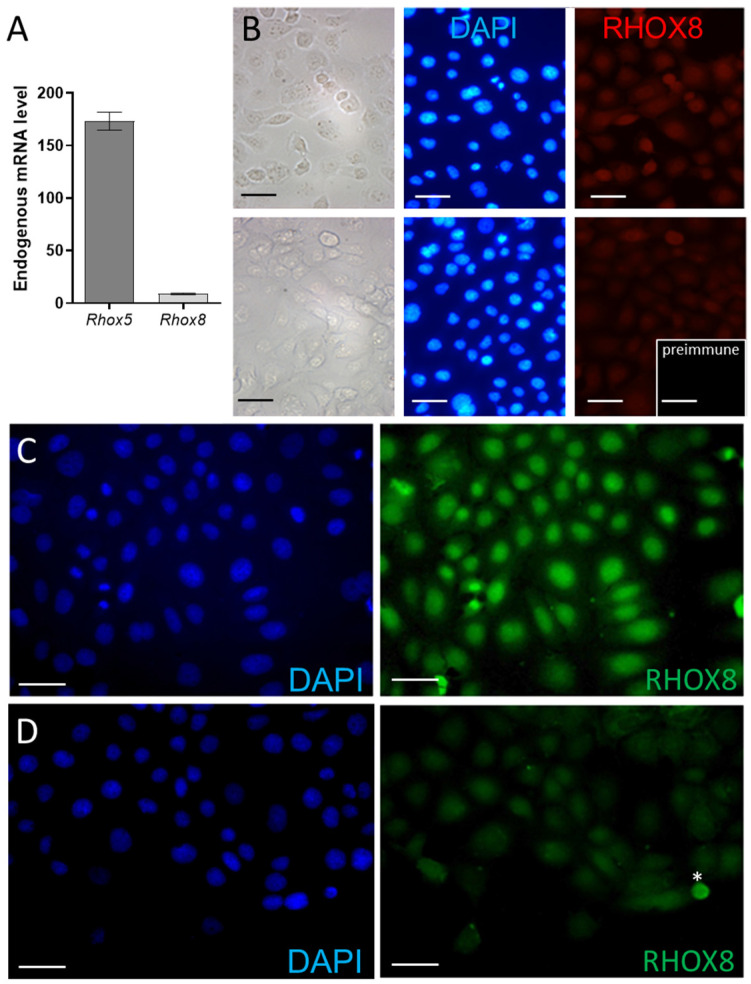
Generation of a stable RHOX8-expressing SIGC line. (**A**) The endogenous levels of rat *Rhox5* and *Rhox8* were determined in parental SIGC cells by qPCR (*n* = 6). (**B**) SIGC cells lack robust nuclear RHOX8 expression as assessed by immunofluorescence in two independent SIGC cultures. (**C**) The stable integration of *Rhox8* transgene into the flip-in site of LacZeo competent SIGC cells resulted in uniform abundant nuclear expression of RHOX8. (**D**) The expression of RHOX8 could be inhibited by the addition of a *Rhox8* siRNA cocktail. The non-specific staining of a dying GC lifting off the plate is indicated by the asterisk (*). Scale bars = 50 µm.

The promoter region responsible for driving *Ins2* expression in the rat pancreas was previously characterized [40]. We used modified versions of this *Ins2* reporter plasmid to characterize the RHOX regulation of *Ins2* in mouse and rat Sertoli cells [16]. Sertoli cells are the analogous male counterpart to female granulosa cells in that they nurse germ cell development. Thus, we reasoned that RHOX factors might regulate *Ins2* in granulosa cells and chose the SIGC line for our in vitro analyses. SIGC cells lack robust *Rhox8* mRNA expression (Figure 3A) and nuclear RHOX8 protein is barely detectable in parental SIGC cultures (Figure 3B). In contrast, endogenous *Rhox5* is highly expressed in SIGC (Figure 3A). In agreement with mRNA data, the RHOX8 protein was not detectable in two independent parental SIGC cultures (Figure 3B). To employ SIGC cells as a tool for the analysis of RHOX8-regulated factors, we generated a stable *Rhox8*-overexpression SIGC line that exhibited a strong nuclear expression of RHOX8 (Figure 3C). This exogenous expression of RHOX8 could be knocked down to endogenous levels (or lower) by the transient transfection of an *Rhox8* siRNA inhibitory cocktail (Figure 3D).

**Figure 4:**
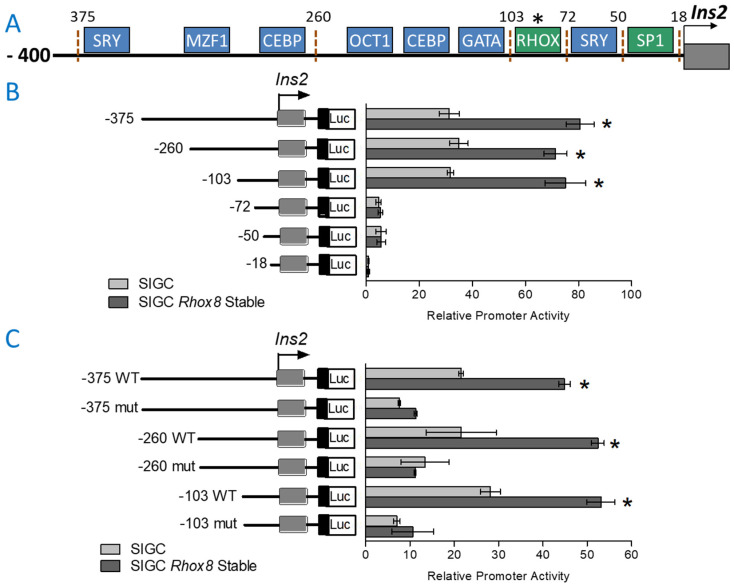
RHOX8 drives maximal *Ins2* promoter activity in SIGC cells. (**A**) Putative transcription factor binding sites contained within the *Ins2* promoter were determined by TESS and TFSEARCH algorithms. Putative positive regulators of *Ins2* expression are indicated in green from prior studies [16,35,43,44] and factors of unknown relevance but established granulosa expression in blue. (**B**) Relative luciferase activity from *Ins2* promoter constructs transiently transfected rat SIGC granulosa cells. Values shown are the ratios of *Ins2* promoter-dependent firefly luciferase activity normalized by *Renilla* luciferase activity internal control (pRL-TK) for transfection efficiency and expressed as fold above pGL3-basic vector, which was arbitrarily assigned a value of 1. Assays were performed in triplicate with three independent preparations of SIGC- and *Rhox8*-stable cell clones. Data are shown as mean ± SEM relative promoter activity and significant differences determined by unpaired *t*-test, * *p* < 0.001. (**C**) Promoter activity in constructs with mutation in the putative homeobox binding site (*RHOX) were assayed as in panel (**B**).

To identify the specific promoter elements responsible for the regulation of *Ins2* expression in granulosa cells, we examined the established 375 nucleotide (nt) *Ins2* promoter for putative activating transcription factors known to be expressed in the ovary (Figure 4A). We then generated a series of deletion constructs from the full-length promoter luciferase reporter that systematically eliminated these potential *Ins2* drivers and assessed residual activity after transfection into either parental SIGC cells or those that overexpressed RHOX8 from the stable transgene. We discovered that the elimination of the putative transcription factor binding element between 357 nt and 103 nt upstream of the transcription start site did not alter the maximal activity of the *Ins2* reporter construct (Figure 4B). SIGC cells with elevated RHOX8 exhibited a 4-fold increase in *Ins2* promoter activity relative to parental SIGC cells. However, shortening the promoter to 72 nt, which eliminated the RHOX binding site we previously characterized in Sertoli cells [16], resulted in substantial downregulation of *Ins2* promoter activity. The SP1 transcription factor is known to stimulate gene expression, including that of *Rhox5* [35], in granulosa cells and further truncation of its binding site reduced reporter activity to background levels (Figure 4B). The putative homeobox binding site contains a consensus CTTAATG core binding site that, when mutated to CTccATG in the context of the 103, 260, and 375 nt promoters, resulted in diminished activity (Figure 4C). When this “RHOX” binding site was intact, the promoter activity was ~2–3 fold higher in parental SIGC cells and 5-fold higher in stable *Rhox8*-expressing cell lines, indicating that homeodomain transcription factors are a major driver of maximal *Ins2* expression in granulosa cells.

**Figure 5:**
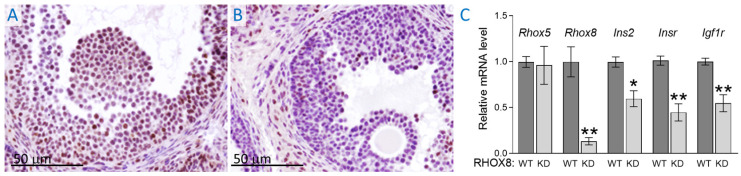
In vivo knockdown of RHOX8 results in reduced expression of insulin signaling in mouse granulosa cells. (**A**) The expression of RHOX8 in mural granulosa cells was assessed in TARGATT transgenic mice without Cre-dependent activation of the in vivo siRNA. (**B**) Activation of the transgene by *Amhr2*-Cre resulted in knockdown of RHOX8 in the majority of mural granulosa cells. (**C**) Mice were induced to superovulate and relative gene expression was determined at 8 h post-hCG as determined in Figure 1. Data are shown as mean ± SEM relative mRNA expression levels and significant differences determined by unpaired *t*-test, * *p* < 0.01, ** *p* < 0.001.

To determine if RHOX8 might activate *Ins2* transcription in mouse granulosa cells, we created an inducible *Rhox8* shRNA transgenic mouse (with the same validated targeting sequence shown in Figure 3D) that could be conditionally activated using Cre/Lox. These mice utilized Applied Stem Cells’ TARGATT system [45,46]. We chose this system because we had previously shown that RHOX8 could be knocked down in vivo by RNA interference (RNAi) [36], and RNAi transgenes can act in a dominant fashion from a random genome integration site. This is necessary to examine *Rhox5/Rhox8* double knockouts as the two genes are only 40 kb apart on the X chromosome and the breeding of two single knockouts to eliminate both genes was unlikely. The *Rhox8* targeting sequence was driven by the ubiquitous U6 Pol III promoter [47,48], which is activated by the CRE recombinase removal of a stop cassette leading to transcription of the *Rhox8*-shRNA transgene and RHOX8 knockdown. We used the anti-Müllerian hormone type 2 receptor (*Amhr2*)-Cre to activate the RNAi transgene as it turns on in granulosa cells when *Rhox5* and *Rhox8* are initially induced [49]. The *Amhr2*-induced shRNA was effective and led to a 7-fold reduction in *Rhox8* mRNA levels (Figure 5) in total RNA prepared from whole ovaries collected at 8 h post-hCG. However, *Rhox5* mRNA levels were unchanged in RHOX8-KD mice, suggesting the knockdown effect was specific. The expression of *Ins2* was diminished 2-fold after knockdown of RHOX8. We have recently examined the conditional ablation of the insulin receptor encoding genes *Insr* and *Igf1r* with *Amhr2*-Cre [50] or *Pgr*-Cre [41] and found that the loss of insulin signaling results in suboptimal ovulation. Thus, we were curious if RHOX8 might regulate these receptors in vivo. The in vivo knockdown of RHOX8 resulted in a significant decrease in both *Insr* and *Igf1r* mRNA expression (Figure 5). However, whether the ~2-fold decrease is sufficient to alter insulin signaling enough to impact successful ovulation was not determined as a formal breeding analysis was not conducted for these mice.

## 4. Discussion

In the present study, we examined the regulation of the primary rodent insulin hormone gene *Ins2* by the two members of the *Rhox* gene cluster that are expressed in ovarian granulosa cells, *Rhox5* and *Rhox8*. To date, the majority of RHOX studies have focused on male reproduction, mostly due to the fact that *Rhox5* is a hallmark androgen-regulated gene that is used as a readout by many endocrinology labs and the desire to determine pathways that underly the spermatogenesis defects observed in *Rhox5*-null mice [23]. We previously demonstrated that in rodent Sertoli cells, RHOX5 is the primary driver of *Ins2* expression and it is one of the few direct targets for RHOX factors that has been validated by chromatin immunoprecipitation [16]. The Akita mouse, which has a similar subfertility phenotype in males as *Rhox5*-null mice, harbors a mutation in the INS2 protein that develops systemic diabetes that, in turn, affects fertility [17,51]. Thus, it has been our expectation that disrupted INS2-dependent signaling in our knockout mice has been a primary contributor to the germ cell loss in the testis and, potentially, motility defects arising in the testis or epididymis, which we have previously described [16,37]. However, the Akita mouse also exhibits severe defects in ovulation [52] that were not found in *Rhox5*-null mice, suggesting that if *Ins2* expression locally in the ovary is essential for ovulation, there must be additional factors that govern its expression.

Our in vitro and in vivo analyses herein indicate that RHOX8 could be one of these factors. The SIGC line we used for our promoter analyses was developed from primary rat ovarian preantral granulosa cells and is a good model to examine granulosa cell differentiation [42]. It mimics the stage of granulosa cell development where endogenous *Rhox5* transcription occurs. It has the established signaling pathways for endogenous *Rhox5* regulation as well as FSH, LH, and estrogen signaling axes [35,53]. SIGC cells were immortalized by the overexpression of TRP53, which activates *Rhox5* in most tumor cells lines, regardless of their tissue of origin [26,27,54,55]. Thus, it was not surprising to find high endogenous *Rhox5* expression. The SIGC line has been shown to maintain granulosa cell-like features even upon multiple passages and does not form tumors in soft agar assays [35,53]. Initially, RHOX5 may be able to stimulate *Ins2* in the absence of RHOX8. In support of this, SIGC cells that lack endogenous RHOX8 have abundant expression of *Rhox5*, and parental SIGC cells do exhibit robust activation of the *Ins2* promoter when the homeobox protein binding site (72 to 103 nt upstream of the *Ins2* transcription start site) that we have deemed the “RHOX” binding site is present. This suggests that in the absence of RHOX8, RHOX5 may supply at least basal *Ins2* activation, but RHOX8 is a more potent activator of *Ins2* in granulosa cells. It is possible that RHOX8 may synergize with RHOX5 to increase activation to maximal levels, or alternatively, RHOX5 and/or RHOX8 may induce the expression of another homeodomain-containing transcription factor that binds this element and maintains *Ins2* expression throughout ovulation. While the putative regulation of *Ins2* by a homeobox factor is likely true in vivo, the direct role of RHOX8 or another specific homeobox transcription factor cannot be validated without chromatin immunoprecipitation with a high-quality antibody. Given the timing of expression, it cannot be ruled out that LH or progesterone could directly influence the expression of *Ins2* in vivo. However, it is unlikely that *Ins2* is a direct target of progesterone receptors, as the reporter construct is highly active in SIGC cells that lack nuclear progesterone receptors (PGR), although a role of the membrane-bound receptor isoform can not be ruled out. Indeed, the lack of nuclear PGR [56] is likely why the parental SIGC lack endogenous *Rhox8* expression, as we previously identified *Rhox8* as a direct target of PGRA [33].

In our prior report, we hypothesized that testes generate local insulins to either circumvent potential blockade by the blood–testis barrier or, more likely, to keep spermatogenic output at a maximum when the dietary state of males is of poor quality. However, it is a mystery why female mice would require local insulin, as there is no barrier to oocyte access from the granulosa cells. Further, women with poor metabolic state have pathways in place to limit conception and implantation when there is not enough energy to support a successful pregnancy [1,57,58], so the granulosa cell production of INS2 that could override this mechanism would seem counterintuitive. The potential existence and role of a conserved RHOX regulation of insulin production in human granulosa cells is uncertain. Most lines of evidence suggest that a somatic cell-specific RHOX factor may not be present in the human ovary and that it is restricted to oocytes [29]. However, human granulosa cells do transcribe and secrete many players in the insulin-like growth factor signaling cascade that act locally in a paracrine fashion within growing follicles to support folliculogenesis and ovulation [59,60,61].

While human granulosa cells may not make insulin protein locally, it is certain that INS acting via its cognate receptor INSR is important for LH responsiveness in human granulosa cells from normal and pathological ovaries [57,58], and the importance of insulin signaling has been validated in many immortalized human granulosa in vitro studies [62,63,64,65]. The insulin regulation of steroidogenesis and proliferation of granulosa cells has been well established in cattle [66], and insulin signaling is required for fertilization competent oocytes in vivo and in vitro [62,67,68,69]. In addition to the control of glucose availability in ovarian cells [70], insulin signaling through its cognate receptors is known to initiate signaling cascades responsible for many developmental programs [71]. Thus, the downstream events regulated by insulin receptors may well be conserved between rodents and humans. To model these processes in mice, we have begun to dissect the insulin-dependent pathways that are downstream of INSR and IGF1R using conditional knockout models that preserve insulin secretion systemically (i.e., the mice are not diabetic) but lack the ability to respond to insulin ligands in granulosa cells. In the first report [41], we used Pgr-Cre, which resulted in the ablation of insulin receptors primarily in the same periovulatory window where we observed *Ins2* expression. The fertility complications of these mice were primarily due to the dysregulation of uterine proliferation and implantation failure [72]. However, mice lacking both INSR and IGF1R had a 50% reduction in ovulation, with oocytes becoming trapped in corpora lutea [41].

Progesterone production by these abnormal corpora lutea was diminished, which suggests that INS2 might serve as part of a feedback loop in response to progesterone-regulated *Rhox8* indicating that folliculogenesis is proceeding normally. We have similarly used *Amhr2*-Cre to ablate *Insr* and *Igf1r* in the granulosa cells of follicles transitioning from the pre-antral to the antral stage [50]. We found that Amhr2-Cre was insufficient to elicit a significant impact on ovarian function, as ovulation rates were normal and the mice exhibited typical estrous cycles [50]. Immunohistochemistry for both receptors indicated that mosaic expression was present at the time of ovulation. We are not certain if that was due to suboptimal or non-uniform activity of the Cre or whether INSR and IGF1R that were present in secondary follicles at the time of *Amhr2*-Cre activation simply did not turn over until follicles reached the ability to skip the block in antral formation. The half-life of insulin receptors in ovarian cells has not been examined, but it is likely that both factors may contribute to the minor impact on ovulation. In this report, we used *Amhr2*-Cre to activate an inhibitory transgene to knockdown RHOX8. We did observe a ~50% decline of *Ins2*, *Insr*, and *Igf1r* in total ovarian RNA from knockdown ovaries. This provides further evidence that the RHOX8 regulation of *Ins2* expression we found in our promoter analysis may be relevant in vivo. The potential co-regulation of INS2 receptors is also interesting, as RHOX8 may optimize the ability of follicles to respond to growth-promoting and differentiation-promoting insulin-dependent factors. We did not formally quantitate residual RHOX8 expression in this model, but ~20% of mural granulosa cells still had nuclear RHOX8 at 8 h post-hCG, which matches the amount of *Rhox8* mRNA that could still be detected by qPCR, and in the future, we will examine *Rhox8*-null mice for the dysregulation of these genes with enhanced rigor.

## 5. Conclusions

In contrast to male mice, where RHOX5 is a primary driver of *Ins2* expression in the testis, we found that while *Rhox5* may be permissive for INS2 secretion, it is RHOX8 that is the likely primary driver of *Ins2* expression in granulosa cells of the rodent ovary.

## Figures and Tables

**Table 1:** Primers for qPCR.

Gene		Sequence of Forward and Reverse Primers 5′-3′	Amplicon Length (bp)	TM
*Rhox5*	For	GCCTGGGAGTCAAGGAA	187	60°
	Rev	AGGACCAGGAGCACCAGGA		
*Rhox8*	For	CCTCAAGAAGTCACCCAGTCG	191	60°
	Rev	ACCTGCGTTCTCCTCTCTCT		
*Ins2*	For	CCTGCTGGCCCTGCTCTT	214	60°
	Rev	CAAGGTCTGAAGGTCACCTG		
*Insr*	For	GCCCTAAGGTCTGCCAAATC	114	60°
	Rev	CTCGGATGTTGATGATCAGGCT		
*Igf1r*	For	GGAGTGTCCCTCAGGCTTCA	217	60°
	Rev	GTTCTCCAACTCCGAGGCAA		
*Rpl19*	For	TGCCTCTAGTGTCCTCCGC	237	60°
	Rev	ATCCGAGCATTGGCAGTACC		

## Data Availability

The original contributions presented in the study are included in the article, further inquiries can be directed to the corresponding author (J.A.M.II).

## Abbreviations

The following abbreviations are used in this manuscript:

SIGC	Spontaneously immortalized granulosa cells
PGR	Progesterone receptor
LH	Luteinizing hormone
qPCR	Quantitative real-time reverse transcription polymerase chain reaction
